# Percutaneous Microcoil Localization of a Small, Totally Endophytic Renal Mass for Nephron-Sparing Surgery: A Case Report and Literature Review

**DOI:** 10.3389/fonc.2022.916787

**Published:** 2022-07-12

**Authors:** Tianhao Su, Zhiyuan Zhang, Meishan Zhao, Gangyue Hao, Ye Tian, Long Jin

**Affiliations:** ^1^ Department of Interventional Radiology, Affiliated Beijing Friendship Hospital, Capital Medical University, Beijing, China; ^2^ Department of Urology Surgery, Affiliated Beijing Friendship Hospital, Capital Medical University, Beijing, China

**Keywords:** microcoil, preoperative localization, partial nephrectomy, endophytic renal mass, computed tomography

## Abstract

Small, totally endophytic renal masses present a technical challenge for surgical extirpation due to poor identifiability during surgery. The method for the precise localization of totally endophytic tumours before nephron-sparing surgery could be optimized. An asymptomatic 70-year-old male presented with a right-sided, 16-mm, totally endophytic renal mass on computed tomography (CT). CT-guided percutaneous microcoil localization was carried out prior to laparoscopy to provide a direction for partial nephrectomy. During the 25 minutes of the localization procedure, the patient underwent five local CT scans, and his cumulative effective radiation dosage was 5.1 mSv. The span between localization and the start of the operation was 15 hours. The laparoscopic operation time was 105 minutes, and the ischaemia time was 25 minutes. The postoperative recovery was smooth, and no perioperative complications occurred. Pathology showed the mass to be renal clear cell carcinoma, WHO/ISUP grade 2, with a 2-mm, clear surgical margin. The patient remained free of recurrence on follow-up for eleven months. To our knowledge, this application of microcoil implantation prior to laparoscopic partial nephrectomy towards an intrarenal mass could be an early reported attempt for the localized method applied in renal surgery. The percutaneous microcoil localization of endophytic renal tumours is potentially safe and effective prior to laparoscopic partial nephrectomy.

## Introduction

Small, totally endophytic renal masses present a technical challenge for surgical extirpation due to poor identifiability during surgery. There are dozens of technology-enhanced methods for tumour localization in several surgical specialities (1–5). However, the method for the precise localization of totally endophytic tumours before nephron-sparing surgery could be optimized. Here, we report the application of percutaneous microcoil localization in a case of a small, totally endophytic renal mass prior to nephron-sparing surgery.

## Case Report

A 70-year-old male was referred to our hospital in June 2020 because of a right renal mass accidentally detected on computed tomography (CT). There were no complaints of haematuria, abdominal masses, pain, or weight loss. The patient’s past medical history included hypertension, coronary artery disease, and chronic hepatitis B. His medications included olmesartan, aspirin and entecavir. There was no family history of cancer, and the physical examination was unremarkable.

**Figure 1:**
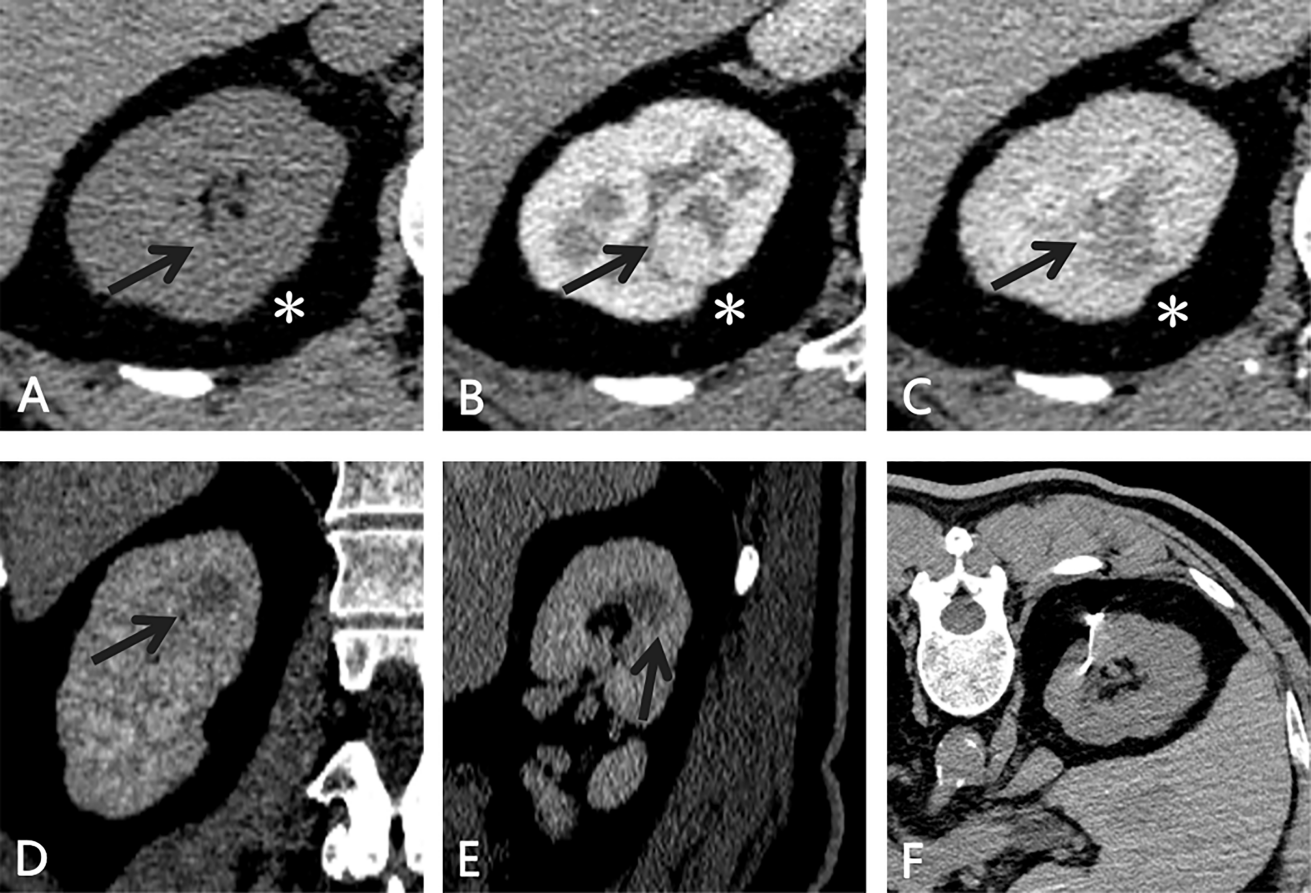
CT of the small, totally endophytic renal mass. **(A–C)** An enhancing endophytic mass(black arrow) measuring 1.6×1.4 cm was observed in the middle part of the right kidney. Slight shrinkage (*) was noted and taken as a puncture reference. During the excretory phase, the mass (black arrow) showed a different enhancement pattern (contrast washout) on coronal **(D)** and sagittal **(E)** views. **(F)** After the microcoil was implanted targeting the lesion, abdominal CT was performed to confirm the position of the microcoil and search for any complications.

Abdominal contrast-enhanced CT revealed a round-like mass with obvious early enhancement in the middle part of the right kidney. The mass did not protrude beyond the renal contour and was accompanied by slight shrinkage of the renal cortex (**Figures 1A–E**). For the purpose of partial nephrectomy, 8 and 4 points were evaluated according to PADUA and SPARE nephrometry scoring systems, respectively (6, 7).

In preoperative discussion, urologists did not believe that the small renal mass could be accurately identified or palpated during nephron-sparing surgery. Considering that the mass was located in the lateral region of the right kidney and intraoperative ultrasound (US) was highly dependent on the operator, we decided to perform microcoil implantation prior to laparoscopy to provide a direction for partial nephrectomy.

After preoperative evaluations were performed and informed consent was obtained, the patient underwent CT-guided localization of the small renal mass without any complications or obvious inconvenience. A tornado-like radio-opaque microcoil (MWCE-18S-6/2-TORNADO, Cook Medical, Bloomington, IN, USA) was implanted into the renal parenchyma targeting the small renal mass (**Figure 1F**).

**Figure 2:**
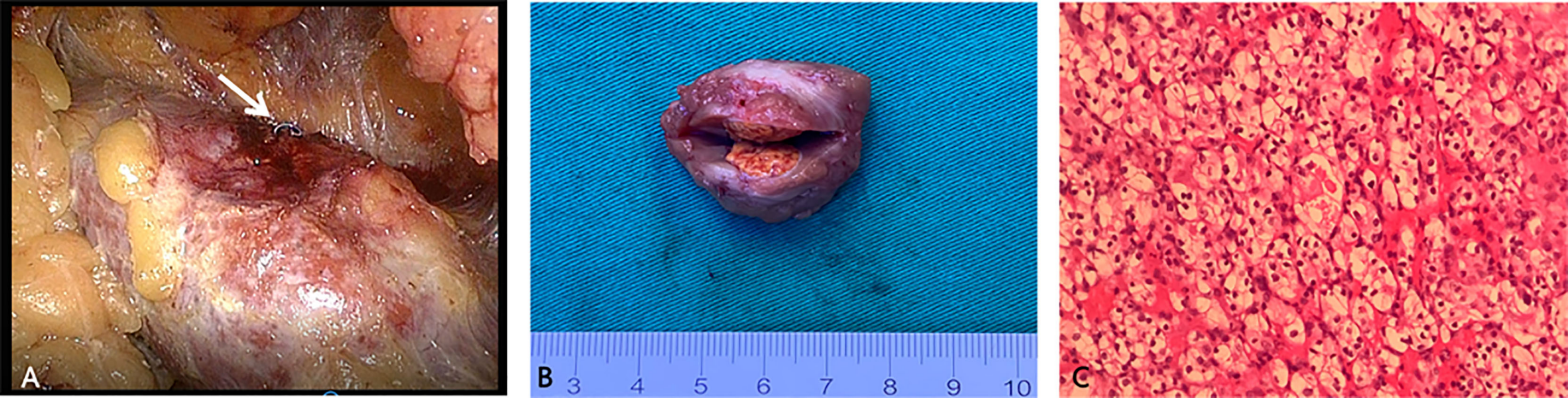
Representative images showing the main steps in video-assisted laparoscopy surgery for the resection of small, totally endophytic renal mass localized preoperatively with the microcoil. **(A)** The microcoil tail (white arrow) was visualized during laparoscopy. **(B)** The excised specimen after partial nephrectomy showed an intact tumour with a clear margin. **(C)** Microscopic sections of the mass showed large polygonal cells with clear cytoplasm and centrally placed small nuclei, indicating renal clear cell carcinoma (haematoxylin-eosin, original magnification 200×).

The right kidney was scanned by CT five times, and the cumulative effective radiation dosage was 5.1 mSv. The span between CT-guided microcoil localization and the start of the operation was 15 hours. Under general anaesthesia, the patient underwent laparoscopic partial nephrectomy in the left lateral position. Laparoscopic exploration of the retroperitoneal cavity revealed the implanted microcoil protruding from the dorsal surface of the right kidney (**Figure 2A**). The resection range was 2 cm in radius with the implanted microcoil as the centre. The mass was completely removed without excessive damage to the renal vasculature or collecting system (**Figure 2B**). The operation time was 105 minutes, and the warm ischaemia time was 25 minutes.

The postoperative recovery was smooth, and no perioperative complications occurred. The postoperative serum creatinine concentration was 88.4 μmol/L, within the normal range, and no hydronephrosis was observed on US at the postoperative follow-up. The final pathological result revealed T1a renal cell carcinoma (RCC, American Joint Committee on Cancer TNM stage), WHO/ISUP grade 2 (**Figure 2C**) (8), 16 mm in greatest dimension. The tumour did not involve the perirenal capsule and was at least 2 mm away from the surgical margin. Eleven months later, follow-up magnetic resonance imaging showed no RCC recurrence, and the patient remained asymptomatic.

## Discussion

The incidence of RCC is rising in large part due to increased utilization of imaging (9). Asymptomatic, localized T1a RCC (≤ 4.0 cm) constitutes the majority of new diagnoses (10). Although several options including partial or radical nephrectomy, ablative therapies and active surveillance could be considered for this patient, partial nephrectomy has a priority because it is fundamental solution, along with his acceptance for surgery and sufficient renal function outcome (11, 12). A prospective, but not a randomized controlled, study concluded a comparative effectiveness of active surveillance with other options. However, active surveillance was intentionally applied in older patient with increased comorbidities (13), which was likely contradicted with surgery. In clinical practice, when it talks about nephrectomy, our preoperative localization can provide a clear direction during laparoscopic exploration and an adequate preservation of renal function after partial nephrectomy.

Nephron-sparing approaches are the recommended and preferred treatment for these patients. Regarding T1a RCC, endophytic tumours are centrally located and nearer to the collecting system (14). Small, totally endophytic renal masses pose some difficulties in terms of laparoscopic nephron-sparing excision, especially in tumour identification and complete resection (2).

A small, totally endophytic renal mass cannot be accurately detected by conventional intraoperative observation or palpation. Intraoperative ultrasound probe is the conventional option for those tumours (15). This is also the case for the up-to-date robot-assisted procedure, so as to score the resection area on mass. However, its application depends on echoic difference from surrounding tissue and subjective operator’s experience (16, 17). Although near-infrared fluorescence imaging has been considered transiently helpful in identifying the vascular anatomy, it is not accomplishable at all for endophytic tumours. The preoperative superselective transarterial delivery of a lipidol-indocyanine green (ICG) mixture to trace endophytic tumours has been described (3, 18), but related transarterial liquid diffusion and allergy must be taken into account. Additionally, intraoperative real-time localization has greater prospects for development; however, this method is not currently widely applied due to the high cost and prolonged operation time (4). It is understandable that 3D models may help in characterizing tumor (19), but 3D virtual model reconstruction still needs a rigorous and acknowledged methodology for clinical practice (20). There have also been case reports on the application of hook-wire localization prior to laparoscopic partial nephrectomy for an intrarenal mass (5, 21, 22); nevertheless, persistent pain is problematic.

We have previously described a modified microcoil method for the precise preoperative localization of pulmonary nodules before video-assisted thoracoscopic surgery, with satisfactory results (23). We adopted a similar method in renal surgery to localize the renal mass. To our knowledge, this application of microcoil implantation prior to laparoscopic partial nephrectomy towards an intrarenal mass could be an early reported attempt for the localized method applied in renal surgery.

Our application provided the opportunity to avoid the use of intraoperative US or an alternative to US if it was not available. This method provides direct guidance in particularly tricky cases of endophytic tumours in which the surgeon requires confirmation of which strategy is best to achieve a safe operation for the patient.

There have been few published reports on the usefulness, efficacy and safety of the microcoil localization of tumours in nephron-sparing surgery. Evidence from video-assisted thoracic surgery suggests that microcoil localization is an effective and useful technique (24–27). The patient in our report received an acceptable radiation dosage and experienced no discomfort. Microcoil localization could be a feasible and safe method that can be used preoperatively to provide enhanced insight into renal masses for urologists. Importantly, our method enables the tail of the microcoil to be easily placed outside the kidney surface. Consequently, the mass can be easily found during laparoscopy, reducing the time required for mass excision. The microcoil, which is usually used for blood vessel embolization, is preloaded and covered by synthetic fibres. These synthetic fibres are intended to activate coagulation and thus may also prevent puncture-related bleeding.

Notably, implantation of the microcoil does not need to be performed on the day of surgery, which is different from approaches using dyes or contrast agents. Concerning hook-wire localization, the introduction of the wire was carried out just before the surgery to minimize the potential risk of wire migration. Therefore, our preoperative localization method is more convenient than others and does not need special equipment or additional time on the day of surgery. After the microcoil was successfully implanted into the renal parenchyma towards the mass, urologists were able to observe the relationship between the microcoil and the endophytic mass on the subsequent CT scans, facilitating the following exploration during laparoscopy.

Although accurately identifying renal masses on CT without contrast perhaps is uneasy sometimes, we could recognize the target mass using CT plane information, for example, nearby blood vessels, bone markers, organs and tissues.As totally endophytic masses are not common among all resectable renal tumours, over the long term, this method could be applied in more cases with similar characteristics to gain more valuable and conclusive results. Possible complications would likely be minor including mirocoil displacement, bleeding, and pain. It is also expected that this microcoil localization with robot-assisted partial nephrectomy would further improve their application for endophytic masses (28). Microcoil localization may also assist performance of tumor enucleation which can be more difficult for entirely endophyritc renal tumors requiring specialized surgical maneuvers (29, 30).

We have reported our experience with the application of microcoil localization for an intrarenal mass. The percutaneous microcoil localization of endophytic renal tumours is potentially safe and effective prior to laparoscopic partial nephrectomy.

## Data Availability Statement

The original contributions presented in the study are included in the article/supplementary material. Further inquiries can be directed to the corresponding author.

## Ethics Statement

The studies involving human participants were reviewed and approved by Clinical Research Ethics Committee of the Beijing Friendship Hospital, Capital Medical University. The patients/participants provided their written informed consent to participate in this study. Written informed consent was obtained from the individual(s) for the publication of any potentially identifiable images or data included in this article.

## Author Contributions

TS and ZZ contributed equally to this work. LJ designed the research. GH and YT performed the surgery. TS, ZZ and MZ analyzed the data. TS and ZZ wrote the paper. All authors read and approved the final manuscript. All authors contributed to the article and approved the submitted version.

## Funding

Beijing Friendship Hospital High-value patent Cultivation Program (2021-03)

## Conflict of Interest

The authors declare that the research was conducted in the absence of any commercial or financial relationships that could be construed as a potential conflict of interest.

## Publisher’s Note

All claims expressed in this article are solely those of the authors and do not necessarily represent those of their affiliated organizations, or those of the publisher, the editors and the reviewers. Any product that may be evaluated in this article, or claim that may be made by its manufacturer, is not guaranteed or endorsed by the publisher.

## Abbreviations

CT computed tomography, RCC renal cell carcinoma.
